# Cost of Serum Versus Skin Allergy Testing Among Medicare Fee-for-Service Beneficiaries in the United States

**DOI:** 10.36469/001c.77482

**Published:** 2023-07-28

**Authors:** Kenny Y. Kwong, Yang Z. Lu

**Affiliations:** 1 Department of Pediatrics Los Angeles County, University of California Medical Center, Los Angeles, California, USA; 2 Department of Health Care Administration California State University, Long Beach, California, USA

**Keywords:** allergy testing, specific IgE, skin, parity, cost, Medicare, utilization

## Abstract

**Background:** Testing for allergic sensitization can be achieved similarly via skin or serum specific immunoglobulin E (sIgE) testing, although the costs of each method differ.

**Objective:** This study compared cost and utilization of allergy testing utilizing skin vs sIgE testing and whether equal access (parity) to both testing methods affects overall allergy testing costs among Medicare fee-for-service beneficiaries in the United States.

**Methods:** Allergy test utilization and payment data were analyzed using 100% 2019 Medicare fee-for-service claims data. Beneficiaries with any sIgE test, skin prick test, or intradermal skin test associated with ICD-10 codes of allergic rhinitis, asthma, and food allergy were included. Aggregate and per-beneficiary testing cost, number of allergens tested, and number of allergy-related specialist visits incurred were estimated by the testing patterns of sIgE only, skin prick only, intradermal only, skin prick and intradermal, and sIgE plus prick and/or intradermal. Medicare Administrative Contractors (MACs) with parity for all allergy tests and those which restricted sIgE testing were compared. Multivariate linear regression was performed on the association between testing patterns and each cost and utilization measure, controlling for parity, age, sex, race/ethnicity, and dual-eligible status.

**Results:** We analyzed 270 831 patients and 327 263 allergy-related claims. Total payment for all allergy tests was 71 380 866,including15 903 954 for sIgE tests, 42 223 930forskinpricktests,and13 252 982 for intradermal tests. Beneficiaries receiving sIgE tests had only 1.8 fewer allergist visits than those with skin prick tests only (0.8 vs 2.6). Cost of testing per beneficiary was also lower in sIgE testing only compared with skin prick tests only (161vs247). Multivariable regression results showed per-beneficiary payments for allergy testing were on average $22 lower in MACs with parity compared with MACs without parity.

**Discussion:** Serum specific IgE testing is associated with lower costs and fewer allergy specialist visits compared with skin testing. Insurance coverage with parity toward sIgE and skin testing is associated with lower overall costs of allergy testing.

**Conclusion:** Among Medicare fee-for-service beneficiaries in the United States, sIgE testing may be more cost effective compared with skin testing in the management of allergic disease.

## BACKGROUND

There is often more than one type of testing of equal accuracy in evaluation and management of disease states. Deciding which test to use is ideally based on clinical factors, However, with rising healthcare expenditures in the United States (US), diagnostic testing costs may need to be considered. Accurate reports in regard to payments for different tests will help facilitate these decisions.

Diagnosis of allergic disease involves clinical history and physical examination, followed by diagnostic tests to identify allergic sensitization. This is most often performed using 2 distinct methods: in vivo skin testing and in vitro laboratory-based serology by serum specific IgE (sIgE) antibody immunoassays. The efficacy of skin testing and sIgE methods are generally considered equivalent for assessing allergic sensitization to inhalant and food allergens.^1–3^

National guidelines provide guidance to diagnosis and management of allergic diseases caused by environmental and food allergens. Accurate identification of responsible allergens causing allergy symptoms is important in planning appropriate management such as pharmacotherapy, biologics, allergen immunotherapy, and allergen avoidance. These can result in symptom reduction and even long-term disease remission.^3^ The National Heart, Lung, and Blood Institute’s Guidelines for the Diagnosis and Management of Asthma EPR-3 recommends identification of triggering allergens for the management of persistent asthma.^4,5^ Similar guidelines for treatment of allergic rhinitis and food allergy also recommend avoidance as a primary management approach after identification of suspected allergen triggers.^6–8^

National guidelines stipulate either skin or sIgE tests may be employed for elucidation of sensitizing antigens in the management of a variety of allergic diseases.^5–7^ Multiple studies have shown equivalent efficacy of skin and serological tests in the identification of allergen sensitization and detection of sIgE antibody in allergy history–positive patients.^9,10^ Skin testing is usually performed by specialists such as allergists in the US, while sIgE testing may be ordered by any type of clinician, including primary care providers. Each test has advantages and disadvantages. While skin testing is relatively simple with results available within 20 to 30 minutes, it is inaccurate in patients taking antihistamines or mast cell–modulating medications and those with dark skin tones and can trigger systemic reactions in high-risk patients. Serum specific IgE tests do not require specialist visits to be performed, are not affected by concomitant medications, and pose no safety risks in patients with a history of severe allergic reactions. However, obtaining blood samples may be difficult in patients with poor venous access.^10^ Choice of which specific test to use in management of allergic disease depends on consideration of these advantages and disadvantages by the provider and patient. However, some health plans and payers restrict use of sIgE tests in favor of skin testing.^11^

Payment for allergy testing in the US differs between skin testing and sIgE testing. Skin testing is a reimbursable physician procedure, usually performed by allergy specialists. More than one allergy specialist visit is generally required to administer skin testing for patients who must discontinue interfering medications. Serum specific IgE tests are performed and billed by reference laboratories; therefore, additional physician office visits are not involved. Even though sIgE and skin allergies have comparable accuracies, there have been no studies systematically examining the utilization patterns and cost of these tests for the diagnosis of allergic sensitization in the US, to our knowledge. Thus, we aimed to examine how each of these allergy testing methodologies has been utilized and their associated expenditures among Medicare fee-for-service beneficiaries. While the prevalence of allergic disease is significantly higher in younger adults compared with Medicare beneficiaries (>65 years of age), there is an unmet need to study allergic diseases among these patients as they have traditionally been excluded from many clinical trials and studies.^12^ Further, results from this study will provide rationale for similar investigations into younger age groups.

The primary aim of this study was to compare utilization patterns and costs of sIgE testing vs skin testing in management of allergic diseases in the US using Medicare fee-for-service claims data. A secondary aim was to compare differences in utilization patterns and costs among Medicare Administrative Contractors (MACs) (private health insurers who administer Medicare benefits), which limit the coverage of sIgE testing in favor of skin testing vs those that provided equal access to both tests. We define MACs that allow unrestricted access to both sIgE and skin testing as having parity and those which restrict sIgE testing in favor of skin testing as having non-parity.

## METHODS

### Sample

We utilized the 100% Medicare fee-for-service enrollment and claims data for calendar year 2019 and included all eligible allergy test claims and all beneficiaries associated with the claims for our cross-sectional study. We compared only skin and sIgE testing as these tests are the only ones recommended by national and global practice parameters for use with asthma, allergic rhinitis, and food allergy with similar accuracy.^4–8^ Eligible allergy tests were sIgE test, skin prick test, and intradermal skin test, identified using Current Procedural Terminology (CPT) codes 86003, 95004, and 95024, respectively. To ensure a fair comparison between sIgE and skin allergy testing, we excluded tests for venom hypersensitivity (ICD-10 codes T63.44-T63.46, Z91.030, Z91.038) and drug allergies (ICD-10 codes Z88.0-Z88.9). Skin prick and intradermal tests, rather than sIgE tests, are the preferred first-line tests for these conditions. We also excluded sIgE tests with CPT code 86003 and the aforementioned ICD-10 codes indicated for venom and drug allergies. This is because sIgE test CPT codes do not specify the allergens being tested, such as pollens vs honeybee venom. Additionally, specific CPT codes (95017 and 95018) for skin and intradermal tests related to venom and drug hypersensitivity were excluded. Serum specific IgE test CPT code 86008, along with its modifiers, was excluded as there are no equivalent skin or intradermal tests for sIgE component testing.

It should be noted that we examined total payments for each type of tests, which included Medicare reimbursement, patient out-of-pocket expenses, and any third-party reimbursement (if applicable). This was to reflect total cost burden of each type of testing. The cost of sharing for most services for Medicare beneficiaries is typically 20%.

### Variables

The key outcome variables were average cost on allergy tests at the beneficiary level, total number of allergy-related specialist visits, and average units/allergens tested.

We restricted the cost of allergy testing as payment for the specific procedure(s) only. For skin testing, there may be additional associated costs due to specialist consultation fees and possible follow-up visits to specialists for test interpretation. To ensure a fair and more direct comparison, we did not include those costs because it would be difficult to ascertain similar costs for sIgE testing, which is often done at a lab location with a prior physician prescription, without apparent associations with any specific physician visit in the claims data. Cost was defined as total payments for each type of testing, including Medicare reimbursement, patient out-of-pocket expenses, and any third-party reimbursement (if applicable).

Allergy-related specialists included allergists, otolaryngologists, and pulmonologists. The key predicting variable was the allergy test patterns of the beneficiaries: (1) sIgE test only, (2) skin prick test only, (3) intradermal skin test only, (4) skin prick test and intradermal skin test, and (5) sIgE and any skin test.

Allergy testing coverage policies are not uniform among the MACs. Specifically, MACs such as CGS, NGS, and Palmetto provide equal coverage for sIgE and skin allergy testing (parity). In contrast, First Coast, Noridian, Novitas, and WPS allow coverage for sIgE testing only when skin testing is contraindicated (non-parity). Hence, we also examined whether such coverage differences led to different allergy test use patterns, overall utilization, and spending on allergy testing. We created a dichotomous variable for parity, based on beneficiary state of residence and geographical jurisdictions of the 7 MACs in operation in 2019. This variable indicated whether a beneficiary lived in a MAC with local coverage determinations for parity, or equal coverage, for sIgE and skin testing.^11^ Each MAC’s local coverage determination for allergy tests was available and obtained from the Centers for Medicare & Medicaid Services.

Other covariates included age, sex, race/ethnicity, and dual-eligible status. As dual-eligible enrollees covered by both Medicare and Medicaid are burdened with higher rates of disabilities and socioeconomic challenges, their demographics and healthcare utilization could systematically differ from the rest of the Medicare population. For example, they account for 17% of Medicare fee-for-service beneficiaries, but 33% of traditional Medicare spending.^13^ For this reason, we believe it was important to include dual-eligible status as a factor in the analyses.

### Statistical Analysis

We conducted summary statistics of the study sample stratified by the 5 allergy test patterns. Bivariate and adjusted analyses of the association between the utilization and cost outcomes and allergy test patterns were conducted. In all bivariate analyses, differences were compared using the χ2 test for categorical variables and independent-samples *t*-test for continuous variables. For the adjusted analyses, we applied multivariable linear regression on each of the 4 outcome variables and controlled for the aforementioned predictors and covariates (allergy test utilization patterns, parity, age, sex, race/ethnicity, and dual-eligible status). We also examined whether allergy test patterns, utilization, and cost differed by allergy test coverage parity. Statistical significance was determined at *P*<.05. All analyses were conducted using SAS 9.4 (SAS Institute, Cary, North Carolina, USA).

### Institutional Review Board/Ethics Approval

This study was exempt from the University of Southern California Keck School of Medicine review under Category 4 of 45 CFR §46.101(2)(b) because it was based on existing de-identified secondary data.

## RESULTS

### Sample Characteristics

Our sample consisted of 270 831 beneficiaries and 327 263 allergy-related claims, with 34% men and 66% women. Total payment for all allergy tests was $71 380 866, including $15 903 954 for sIgE tests, $42 223 930 for skin prick tests, and $13 252 982 for intradermal tests (not shown). **Table 1** reports the patient characteristics, stratified by the 5 allergy test patterns. Similar proportions of males and females received skin and sIgE tests. Thirty-five percent of patients in the over-75 age group received sIgE tests, followed by 33%, 30%, and 29% of patients in the under-65, 70 to 75, and 65 to 69–year age groups, respectively. Approximately one-third of beneficiaries received either sIgE only (32%, n = 86 055) or skin prick only (35%, n = 96 058) testing. Close to one-fourth received both skin prick and intradermal testing (24%, n = 64 698), followed by 6% utilizing a combination of sIgE and any skin testing (prick or intradermal) (n = 14 979) and 3% intradermal testing only (n = 9041) (**Table 1**). The utilization of a single type of test was the lowest among white non-Hispanic patients and the highest among Asians. Among white non-Hispanics, 31% received sIgE tests only and 34% received skin prick test only. In contrast, 38% of Asians utilized sIgE tests only and 45% Asians exclusively utilized skin prick tests.

**Table 1.** Characteristics of Medicare Fee-for-Service Beneficiaries by Allergy Test Utilization Pattern in 2019

	**Allergy Test Utilized, n (%)^a^**					
	**All** **(n = 270 831)**	**sIgE Test Only**	**Skin Prick Test Only**	**Intradermal Skin Test Only**	**Skin Prick and Intradermal Skin Test**	**sIgE and Any Skin Test**
Age (y)						
<65	37 880 (14)	12 362 (33)	13 813 (36)	1168 (3)	8261 (22)	2276 (6)
65-69	71 286 (26)	20 760 (29)	24 746 (35)	2344 (3)	19 320 (27)	4116 (6)
70-75	75 752 (28)	22 618 (30)	26 568 (35)	2830 (4)	19 396 (26)	4340 (6)
75+	85 913 (32)	30 315 (35)	30 931 (36)	2699 (3)	17 721 (21)	4247 (5)
Sex						
Male	92 133 (34)	28 876 (31)	33 341 (36)	3031 (3)	22 105 (24)	4780 (5)
Female	178 698 (66)	57 179 (32)	62 717 (35)	6010 (3)	42 593 (25)	10 199 (6)
Race/ethnicity						
White non-⁠Hispanic	210 097 (78)	64 682 (31)	71 356 (34)	7738 (4)	54 709 (26)	11 612 (6)
African American	22 773 (8)	7799 (34)	9232 (41)	521 (2)	3896 (17)	1325 (6)
Hispanic	17 955 (7)	6554 (37)	7209 (40)	360 (2)	2732 (15)	1100 (6)
Asian	9755 (4)	3737 (38)	4371 (45)	123 (1)	1147 (12)	377 (4)
Native American	750 (0)	270 (36)	262 (35)	32 (4)	149 (20)	37 (5)
Other	2613 (1)	931 (36)	1016 (39)	60 (2)	477 (18)	129 (5)
Missing	6888 (3)	2082 (30)	2612 (38)	207 (3)	1588 (23)	399 (6)
Subtotal		86 055 (32)	96 058 (35)	9041 (3)	64 698 (24)	14 979 (6)

^a^Data in the first column (All) are displayed as number (%) for the whole sample; data in other columns by allergy test utilization pattern are displayed by number (%) according to the row.

Abbreviation: SIgE, serum specific IgE test.

### Bivariate Associations

**Figure 1.**
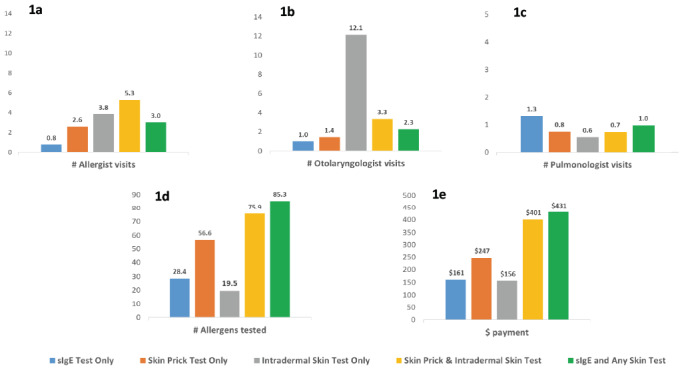
Specialist Visits, Allergens Tested, and Payment per Beneficiary by Test Utilization Pattern (**1a**) Mean total number of visits to allergists; (**1b**) mean total number of visits to otolaryngologists; (**1c**) mean total number of visits to pulmonologists; (**1d**) mean total number of allergens tested; (**1e**) mean total payment. Independent-samples *t*-test was used to compare the differences between sIgE test only and skin prick test only (all *P*<.001). Abbreviation: sIgE, serum specific IgE test.

Compared with patients receiving skin prick test only, those with sIgE test only had fewer allergist (0.8 vs 2.6) and otolaryngologist (1.0 vs 1.4) visits, had more pulmonologist (1.3 vs 0.8) visits, were tested for fewer allergens (28.4 vs 56.6 units), and incurred lower spending on their allergy tests ($161 vs $247), all *P* < .001 (**Figure 1**). Patients with intradermal skin test only had the highest number of otolaryngologist visits (12.1). Patients with both skin prick test and intradermal skin test on average visited allergists 5.3 times, the highest of all groups (**Figure 1**).

**Figure 2.**
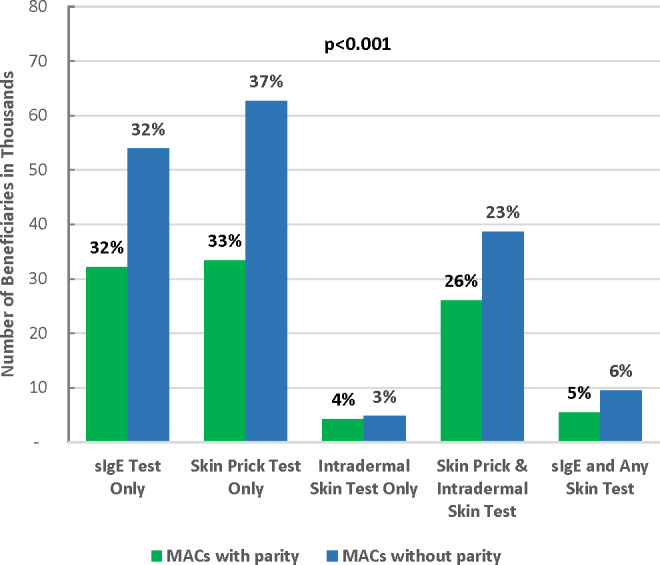
Number and Percentage of Medicare Beneficiaries by Test Utilization Pattern and Parity Status Note: Chi-square test was used to test the dependence between test utilization pattern and parity status. Abbreviation: MACs, Medicare Administrative Contractors.

**Figure 3.**
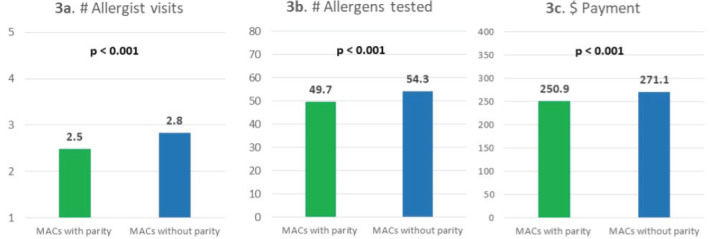
Specialist Visits, Allergens Tested, and Payment per Beneficiary by Parity Status (**3a**) Mean total number of visits to allergists; (**3b**) mean total number of allergens tested; (**3c**) mean total payment. Independent-samples *t*-test was used to compare the differences by parity status. Abbreviation: MACs, Medicare Administrative Contractors.

MACs with parity had a similar percentage of sIgE testing but a lower percentage of skin prick testing compared with MACs without parity. Specifically, among patients in MACs with parity, 32% used IgE test only, 33% used skin prick test only, 4% used intradermal skin test only, 26% used both skin prick and intradermal skin tests, and 5% used sIgE test and any skin test. The percentages were 32%, 37%, 3%, 23%, and 6% for patients in MACs without parity, respectively (**Figure 2**). Test utilization patterns were statistically significantly different between patients in MACs with and without parity (*P*<.001). Overall, parity toward sIgE and skin testing coverage was associated with lower allergy test–related utilization and spending. Specifically, compared with patients in MACs without parity, patients in MACs with parity had fewer allergist visits (2.5 vs 2.8), fewer units of allergens tested (49.7 vs 54.3), and lower payment for all allergy tests per person ($251 vs $271; all *P*<.001) (**Figure 3**).

### Multivariable Linear Regression Results

Multivariable linear regression results showed that demographics were associated with utilization and cost outcomes (**Table 2**). Specifically, those 75 years or older had 0.4 fewer specialist visits, 0.3 fewer allergist visits, and 0.8 more allergens tested, and incurred $6.10 more in payment than those between 65 and 69 years old (all *P*<.001). Females were associated with 0.2 fewer specialist visits, 1.0 more allergens tested, and $5.30 lower payment than males (all *P*<.001). Compared with non-Hispanic whites, racial groups including African Americans, Hispanics, and Asians were associated with more allergens tested and higher payment, although they had fewer specialist visits (all *P*<.001).

**Table 2.** Multivariate Linear Regression Results: Specialist Visits, Allergens Tested, and Payment at the Beneficiary Level

	**Outcome 1: No. of Specialist Visits**		**Outcome 2: No. of Allergist Visits**		**Outcome 3: No. of Allergens Tested**		**Outcome 4: Payment of Testing, $**	
	**Beta**	***P* Value**	**Beta**	***P* Value**	**Beta**	***P* Value**	**Beta**	***P* Value**
Age (y)								
<65	0.1	.268	0.0	.585	-3.2	.000	-16.3	<.001
65-69^a^								
70-75	0.1	.046	0.0	.754	0.1	.569	0.8	.238
>75	-0.4	<.001	-0.3	<.001	0.8	.000	6.1	<.001
Sex								
Male^a^								
Female	-0.2	<.001	0.1	.088	-1.0	<.001	-5.3	<.001
Race/ethnicity								
White non-Hispanic^a^								
African American	-0.6	<.001	-0.3	<.001	4.4	<.001	25.4	<.001
Hispanic	-0.4	<.001	-0.1	.260	4.4	<.001	29.2	<.001
Asian	-0.5	<.001	0.2	.033	3.9	<.001	34.8	<.001
Native American	-0.5	.126	-0.7	.006	-4.9	<.001	-30.8	<.001
Other	-0.2	.274	0.2	.200	2.2	<.001	22.1	<.001
Missing	0.0	.816	0.3	.002	1.7	<.001	17.8	<.001
Dual-eligible								
Yes	-1.4	<.001	-1.0	<.001	7.7	<.001	43.0	<.001
Parity								
Yes	-0.5	<.001	-0.4	<.001	-4.5	<.001	-22.0	<.001
Test utilization pattern								
sIgE only^a^								
Skin prick only	1.7	<.001	1.8	<.001	28.2	<.001	86.2	<.001
Intradermal only	13.3	<.001	3.0	<.001	-7.1	<.001	5.4	<.001
Skin prick and intradermal	6.1	<.001	4.4	<.001	49.0	<.001	248.5	<.001
SIgE and any skin test	3.0	<.001	2.2	<.001	57.4	<.001	273.7	<.001

^a^Reference group.

Abbreviation: sIgE, serum specific IgE test.

Dual-eligible patients were associated with 1.4 fewer allergy-related specialist visits, 1.0 fewer allergist visits, 7.7 more allergens tested, and $43.00 more in allergy test payments per person (all *P*<.001). Similarly, patients in MACs with parity toward both skin and sIgE testing had slightly lower levels of utilization and cost compared with patients in MACs without parity: they were associated with 0.5 fewer allergy-related specialist visits, 0.4 fewer allergist visits, 4.5 fewer allergens tested, and $22 less in allergy test payments per person (all *P*<.001) (**Table 2**).

Controlling for age, sex, race/ethnicity, dual-eligible status, and parity, patients with skin prick test only were associated with 1.7 more allergy-related specialist visits, 1.8 more allergist visits, 28.2 more units/allergens tested, and $86.20 more in allergy test payments per person than patients with sIgE test only (all *P*<.001) (**Table 2**). Patients who utilized intradermal skin test only or utilized 2 or more types of allergy tests showed similar patterns of higher utilization and higher spending compared with patients with sIgE test only. Notably, holding all else constant, patients who had both skin prick test and intradermal skin test were associated with 6.1 more allergy-related specialist visits, 4.4 more allergist visits, 49.0 more allergens tested, and $248.50 more in allergy test payments per person than patients with sIgE test only (all *P*<.001) (**Table 2**).

## DISCUSSION

This study shows that the direct procedural costs of sIgE testing is lower for the Medicare fee-for-service program and its beneficiaries compared with skin testing in the management of certain allergic diseases (allergic rhinitis, asthma, and food allergy). This is independent of other related clinical and health utilization costs associated with either test. Total payments per beneficiary for sIgE test only was 35% less than for skin prick test only ($161 vs $247), and 60% less for skin prick testing with intradermal testing combined ($161 vs $401). Total payments per beneficiary were similar between sIgE test only and intradermal skin test only (**Figure 1**). MACs with parity for sIgE and skin testing incurred lower costs for all testing modalities compared with those which restricted sIgE testing (**Figure 3; Table 2**).

Skin testing appears to incur higher costs to the Medicare fee-for-service program and its beneficiaries than sIgE testing. For direct payments for testing alone without consideration of physician fees associated with each specific test, skin testing is more costly compared with sIgE testing. This may be due to the fact that skin tests are billed as procedures, which are performed mostly by allergy specialists. Total costs are likely higher as skin tests often require additional allergy specialist visits associated with test interpretation. Serum specific IgE tests may be ordered and interpreted by a wide range of providers including primary care physicians and physician extenders.^10^ In this study, however, the higher cost of skin testing is independent of the associated specialist consultation and/or evaluation fees. It is plausible that the cost difference is related to the slightly higher number of allergens tested per beneficiary in skin tests vs sIgE tests (**Figure 1**).

There have been few studies comparing the costs of sIgE vs skin testing among Medicare beneficiaries in the US. To the best of our knowledge, our paper is the only recent study conducted in the past 20 years. Using a 5% sample of Medicare reimbursements, costs of individual tests, and average number of tests per patient in 1996, Poon et al^14^ projected the average submitted charge for allergen skin testing was lower than sIgE tests ($225 vs $320, respectively. The current study demonstrates that since 1996, this cost difference has flipped, with sIgE testing now costing significantly less compared with skin testing, most likely due to laboratory technology advances over time.^10^ This is supported by the fact that total payments for sIgE tests were lower than skin tests compared with 1996 measures, despite similar percentages of sIgE testing per total number of allergy tests in both studies. There are, however, no comprehensive studies examining trends in allergy specialist procedural fees or reagents for skin testing that may also have contributed to higher costs of skin testing since 1996. Further, Poon et al used submitted Medicare charges on a 5% Medicare sample while our study examined actual payments among 100% Medicare fee-for-service beneficiaries. Therefore, our study reflects actual, up-to-date costs of allergy testing. It is likely that with in vitro allergy testing advances such as micro-array technology, sIgE testing will be even more cost-effective in the near future.

We found that a significantly high percentage of beneficiaries received both skin prick and intradermal testing (24%) (**Table 1**). The latter is more sensitive than the former, but false-positive results are more common with this testing, which also has a higher risk of inducing systemic allergic reactions.^10^ Practice parameters do not specifically recommend against intradermal as first-line tests to determine inhalant allergen atopy, but they are usually employed if initial skin prick testing results are negative.^15,16^ Fifty-nine percent of beneficiaries received some skin prick testing (35% skin prick only plus 24% skin prick and intradermal). Assuming that intradermal tests were performed only among those with negative skin prick test results, 41% of those beneficiaries (24% of 59%) did not react to initial prick testing. While there is a paucity of data on skin prick test reactivity among elderly patients, it is unusual if close to one-third of beneficiaries failed to react to initial skin prick tests.^17^ It is possible, however, that patients who received prick and intradermal tests failed to react to only certain allergens when prick tested and had to be further investigated with intradermal tests. The Medicare claims data do not include information on specific allergens tested or test results, so we were not able to discriminate at this level.

Across all Medicare fee-for-service beneficiaries, sIgE tests were utilized less than skin tests despite being less costly to Medicare (**Figure 1; Table 1**). Broader use of sIgE tests may therefore reduce the cost of overall allergy testing for beneficiaries. We showed that among MACs with equal access to sIgE and skin testing (parity), overall cost of allergy testing is lower compared with MACs that restrict sIgE testing (non-parity). Equal access to different testing modalities likely results in providers and patients choosing tests that are the most cost-effective. Although MACs with parity utilized similar percentages of sIgE tests only compared with MACs without parity, the latter performed higher percentages of skin prick tests only. Our findings showed that higher numbers of allergens were included for skin tests compared with sIgE tests (**Figure 1**). Therefore, it is likely that higher costs incurred by MACs without parity are due to slightly higher numbers of allergens tested, compared with MACs with parity, as shown in **Figure 3b**. It must be emphasized that both sIgE and skin testing should be accessible to patients, because while both tests are of comparable accuracy, they are used in a complimentary fashion in many clinical situations.^10^

Cost savings associated with reduced specialist visits may further be amplified by reductions in future health insurance premiums. As future insurance premiums are calculated based on past claims, reduction of specialist claims associated with sIgE testing may translate to distal premium decreases.

Aside from cost savings, parity to sIgE and skin testing will help address the increasing burden of atopic disease in the US, especially among the elderly. Global shifts to urban lifestyles and microbiome and climate changes have resulted in rising incidence of atopic disease and prolonged exposure of vulnerable individuals to allergens.^18–21^ Compounding this problem is a massive shortage of allergy specialists in the US, which is projected to continuously worsen, resulting in even greater reductions in access and longer wait times for allergy testing. This is especially the case for health plan beneficiaries whose coverage and access for sIgE testing is restricted.^22–28^ These reductions in access to allergy services will continue to disproportionately impact underserved urban and rural areas.^29,30^ The unmet needs of more patients needing allergy testing due to specialist shortages and increasing disease prevalence can in part be ameliorated with increased access and use of sIgE tests in primary care settings in conjunction with strategies to increase access to allergy specialists (eg, use of sIgE tests in telemedicine consults with specialists).

Patterns in testing types were generally consistent across ethnicities. Skin testing was performed more than sIgE testing in all ethnicities except for Native Americans. White non-Hispanic patients had the highest utilization of multiple skin test types, while the utilization of only a single type of test was the most prevalent in Asian Americans. Differences in test pattern utilization among different ethnicities may be due to differences in regional test coverage, access to allergy specialists, and individual preferences.

There are limitations to this study, and results must be interpreted with these in mind. First, the spending on allergy testing among Medicare fee-for-service beneficiaries was small compared with total Medicare expenditures. Although Medicare fee-for-service beneficiaries accounted for 62.3% of the total Medicare population in 2019,^31^ the results might not be generalizable to Medicare Advantage enrollees as their utilization patterns could differ. We compared only procedural costs of skin testing vs sIgE testing and did not include other utilization and provider visits specifically associated with allergy testing. For instance, a provider may schedule an extra visit for interpretation of test results. These extra costs may amplify or reduce the difference between costs of skin and sIgE testing. However, these extra costs may be difficult to capture accurately using claims data. To our knowledge, there are no studies measuring these nonprocedural payments involving allergy tests cited in this study. In small studies involving penicillin skin testing (not included in this study), personnel and nonprocedural costs were similar to actual cost of the procedure.^32^ Studies have shown limited validity of diagnosis-based claims in identification of disease states in Medicare and other forms of insurance.^33,34^ This was a cross-sectional study for a 1-year period, and subjects did not have to be continuously enrolled in Medicare. Outcomes such as specialist visits may therefore be affected if they could not be captured during the specific sampling period. Further, if there were specialist visits made prior to allergy testing within the sampling period, they would have been captured. However, attrition of data is probably similar for all testing types, thus making outcomes from different tests comparable. Nevertheless, data from this retrospective study with the aforementioned limitations provides an impetus for future prospective cohort studies to address this issue.

Some patients with a diagnosis of excluded conditions (eg, drug and venom allergy) may have been included in the data as they also had a diagnosis of asthma, allergic rhinitis, or food allergy. Therefore, testing may have been performed for these excluded conditions. State of residence was used for grouping patients into MACs with and without parity. Patients may have seen multiple providers for allergy testing across different MACs during the year. However, in our data, the vast majority of the patients (98%) stayed within their state of residence and MAC for allergy testing. It is likely that any misclassification would have been trivial. In addition to MAC parity differences, beneficiaries may prefer sIgE testing compared with skin tests and therefore influence patterns of test utilization. Providers may also have biases toward skin vs sIgE test. However, we have shown that age, gender, and race/ethnicity were similar across different patterns of test utilization (**Table 1**). The above sampling bias was minimized as the entire US Medicare fee-for-service population was used for allergy test claim analysis, which is a major strength of the study.

In conclusion, procedural costs of sIgE testing are significantly lower compared with skin testing in evaluation of allergic sensitization. MACs that allow equal access to both testing modalities are associated with patterns of testing utilization, resulting in lower costs compared with MACs that restrict sIgE testing. Broader use and improved access toward sIgE testing is likely to alleviate cost burdens to healthcare systems.

### Disclosures

K.Y.K. and Y.Z.L. are consultants for Thermo-Fisher Scientific.
